# Approaching Gravity as a Continuum Using the Rat Partial Weight-Bearing Model

**DOI:** 10.3390/life10100235

**Published:** 2020-10-08

**Authors:** Marie Mortreux, Megan E. Rosa-Caldwell

**Affiliations:** Department of Neurology, Beth Israel Deaconess Medical Center, Harvard Medical School, Boston, MA 02115, USA; merosaca@bidmc.harvard.edu

**Keywords:** gravity, partial weight-bearing, ground-based, rodent, spaceflight, analog

## Abstract

For decades, scientists have relied on animals to understand the risks and consequences of space travel. Animals remain key to study the physiological alterations during spaceflight and provide crucial information about microgravity-induced changes. While spaceflights may appear common, they remain costly and, coupled with limited cargo areas, do not allow for large sample sizes onboard. In 1979, a model of hindlimb unloading (HU) was successfully created to mimic microgravity and has been used extensively since its creation. Four decades later, the first model of mouse partial weight-bearing (PWB) was developed, aiming at mimicking partial gravity environments. Return to the Lunar surface for astronauts is now imminent and prompted the need for an animal model closer to human physiology; hence in 2018, our laboratory created a new model of PWB for adult rats. In this review, we will focus on the rat model of PWB, from its conception to the current state of knowledge. Additionally, we will address how this new model, used in conjunction with HU, will help implement new paradigms allowing scientists to anticipate the physiological alterations and needs of astronauts. Finally, we will discuss the outstanding questions and future perspectives in space research and propose potential solutions using the rat PWB model.

## 1. Introduction

Spaceflight was once viewed as rare and exceptional but is now widely used by scientists and has attracted private investors and companies, thus democratizing space and microgravity. Data gathered from astronauts onboard the Soyuz, space shuttles or Mir missions and the International Space Station (ISS) have been crucial for understanding the consequences of microgravity in space, and upon return on Earth [1,2,3,4,5,6]. Decades of research have established dramatic physiological changes as result of exposure to microgravity to spaceflight, including: reduced muscular mass and strength [7], reduced bone mineral density [8], cardiovascular complications, as well as others [9,10]. Upon returning to Earth, these adaptations can dramatically influence astronaut health and quality of life. Therefore, ground-based models are necessary to understand the full scope of microgravity-induced pathologies as well as interventions to mitigate these while in space.

For centuries, scientists have used animals to better discriminate between physiological and pathological states and investigate human-related diseases and conditions [11,12]. Although Yuri Gagarin was the first human in space, he was preceded by many animals that helped assess risks, feasibility, and outcomes [13,14]. While several species have been flown in space and each can bring valuable information, space missions can be highly restrictive and not easily allow for longitudinal assessments or controlled variables.

As more information was needed to better understand and mitigate astronauts/cosmonauts deconditioning when exposed to microgravity, and ensure their safety after their mission, scientists needed to establish a ground-based model to mimic microgravity and unravel the risks and consequences. Rodents have long been the preferred preclinical model for biomedical research both in space and on Earth [15], and in 1979, Morey described the first model of rat hindlimb unloading (HU) [16], which is now the gold-standard to explore the physiological response to mechanical unloading, an analog model of microgravity. While decades of research into unloading-induced alterations to physiological systems have provided invaluable data on microgravity-induced aberrations to physiology, the standard HU model does not account for the variations of gravity inherent to different astronomical settings, such as the Moon or Mars.

Artemis missions are set to land the first woman and the next man on the Moon in 2024. These missions will set the stage for a lunar base and serve as a stepping-stone for an eventual crewed-exploration to Mars [17,18]. Therefore, to thoroughly prepare for this endeavor, there is an urge to study gravity as a continuum, and develop models mimicking partial gravity environments. The first attempt at simulating partial gravity on earth was attempted in 2010, with Moon (0.16*g*) or Mars (0.38*g*) gravitational loads in female mice [19]. However, inbred mice may be too homogenous to have the desired transferability to human physiology in different gravitational environments. Recently, this model has been adapted to outbred rats in order to better understand the individual variations in response to partial reductions in mechanical loading [20]. Therefore the purpose of this review is to summarize the methodology and utility of this rat partial weight bearing (PWB) model in relation to human physiology, summarize what is currently known on the musculoskeletal effects of partial gravity environments, and provide future directions for the use of this model as it relates to spaceflight and physiology.

## 2. Establishing the Rat Partial Weight-Bearing (PWB) Model

In 2010, the first successful model of quadrupedal partial weight bearing (PWB) was developed in mice to study the impact of reduced mechanical loading on the musculoskeletal system [19]. Ten-week-old nulliparous female BALB/cByJ mice were exposed to 21 days of Mars-analog loading, (38% of normal loading), and compared to jacketed controls (full weight-bearing). This study showed that PWB led to ~20% lower gastrocnemius wet mass and ~24% lower femoral bone volume, and was the first study to demonstrate functional alterations in muscle force production in simulated partial gravity environments. In a later study using female C57Bl/6J mice exposed to various degrees of mechanical unloading [21], the authors confirmed their previous observations and demonstrated that reductions in weight-bearing led to a dose-dependent atrophy of the hindlimb muscles and a decline in bone mineral density (BMD). Since then, this murine model has been used in several ground-based studies to investigate the effects of PWB on blood cell count [22], bone loss mechanisms [23,24,25], and in conjunction with low-dose high linear energy transfer (LET) radiation [26].

The establishment of the mouse PWB model provided a unique opportunity to investigate the consequences of lunar and Martian environments on animal physiology in ground-based studies when other alternatives were not yet available. Until recently, PWB was exclusively used on female inbred mice. However, rats demonstrated to provide potentially more accurate information regarding human physiology [27]. Studies are often carried on outbred strains to better account for the genetic diversity observed in humans, and these animals share many physiological and cognitive characteristics with humans [28,29]. Moreover, rats are smart, trainable [28], and social animals that process drugs similarly to humans [30]. Additionally, their size, approximately twenty times greater for an adult rat compared to a mouse, allows for easier surgical procedures [31] and thorough histopathological analyses. However, this change of scale for the PWB model required technical adjustments that were crucial in order to successfully adapt it for rats. These areas include addressing the housing environment, changing the suspension apparatus, and developing a pelvic harness.

### 2.1. Housing Environment

The housing environment in the rat PWB model was designed to fulfill several requirements [20]. Firstly, it had to provide a floor space large enough for the rat to move around and explore. Secondly, it had to reduce waste accumulation and minimize the components that could be used by the animals to increase mechanical loading. Finally, it had to be of an appropriate size to accommodate the entire suspension apparatus, during either full mechanical loading, complete unloading, or partial weight-bearing, regardless of the animals’ growth, which remains constant between 10 and 30 weeks of age [32].

**Figure 1:**
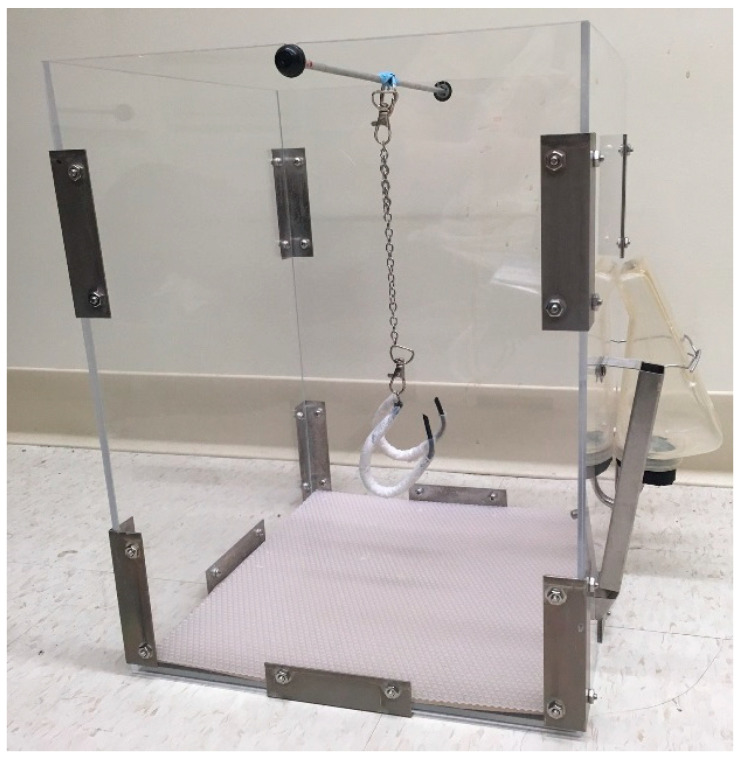
Photograph of the cage designed for the rat partial weight bearing (PWB) model with a suspension apparatus.

The cages were made of five panels of clear polycarbonate with the following dimensions: 12” wide × 12” deep × 16” high (Figure 1). At the top of both side panels, a small centered indentation was made in order to support the suspension apparatus. To avoid waste accumulation, we chose to use a 12” × 12” flexible perforated polypropylene floor panel layered with absorbent paper. This alternative was preferred over wire-floors, which have been shown to promote foot lesions [33] and induce thermal stress [34] in rats. While this type of flooring did not prevent food waste from falling onto the floor, a food chute was installed at the exterior of the cage that allowed only one pellet to be dispensed at the time, thus reducing waste and preventing any opportunities for the rats to reload themselves via the food pellets. Although the housing was first designed without any type of enrichment for the animals, more recent studies are now conducted with the addition of a couple of autoclaved cotton-squares that are changed every other day, so as to improve animals’ wellbeing.

### 2.2. Suspension Apparatus

**Figure 2:**
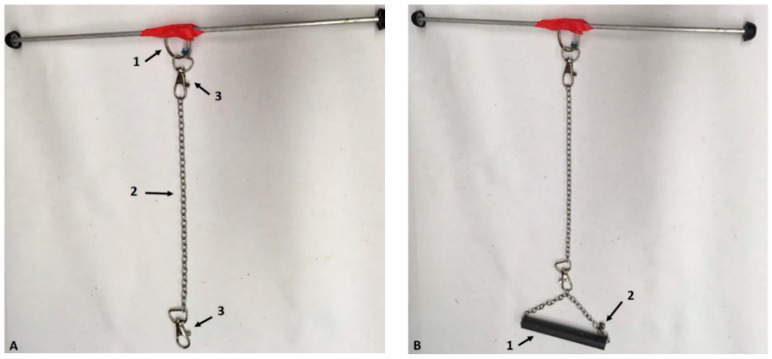
Photographs of the suspension apparatus used for HLU (**A**) and PWB (**B**). Arrows point to key elements in panels (**A**) 1: centered key ring, 2: stainless steel chain, 3: swivel clasps, and (**B**) 1: back rod, 2: clasps. Adapted from [35].

In order to support skeletally mature male rats with a starting body weight of approximately 400 g, many adjustments were required with respect to the suspension apparatus (Figure 2). While rats have enough floor space in the cages according to the Institutional Animal Care and Use Committee (IACUC) guidelines, their larger size allows them to freely move around and explore their environment, even when partially unloaded; therefore, suspension apparatus was secured at the center of the aluminum rod. In essence, the suspension design differed slightly from the one previously used in mice [19,23], retaining a triangle-shape structure linking the forelimb jacket and hindlimb suspension device with a back-rod, improving stabilization. A swivel clasp was added on both ends of the stainless-steel chain to provide full mobility to the animals, allowing them to move around without risking entangling the chains and modifying their partial unloading level. The most critical part of the suspension apparatus underwent major alterations to accommodate rats and improve PWB stability. In mice, a bead chain was positioned by twisting the coils of a spring with known characteristics, allowing animals to gain kinetic energy and increase mobility. This system was not an ideal fit for rats and was replaced by a key-chain ring attached to a swivel clasp directly connected to the stainless-steel chain. This design did not allow for additional kinetic energy, or variations in chain length, henceforth greatly reducing daily variations and the need to do adjustments throughout the experiments.

In order to achieve specific levels of PWB, animals are first weighed while wearing the entire PWB apparatus (100% full weight-bearing). The links on the chain are then adjusted to raise the animals to the appropriate PWB level (Figure 2A, items 2 and 3). Achieved weight-bearing is confirmed using a scale throughout this process. The chains have been selected to allow for steady and fine tuning of weight-bearing, maintaining the desired weight within 5% of error 100% of the time.

### 2.3. Using a Pelvic Harness during Suspension (HLS)

**Figure 3:**
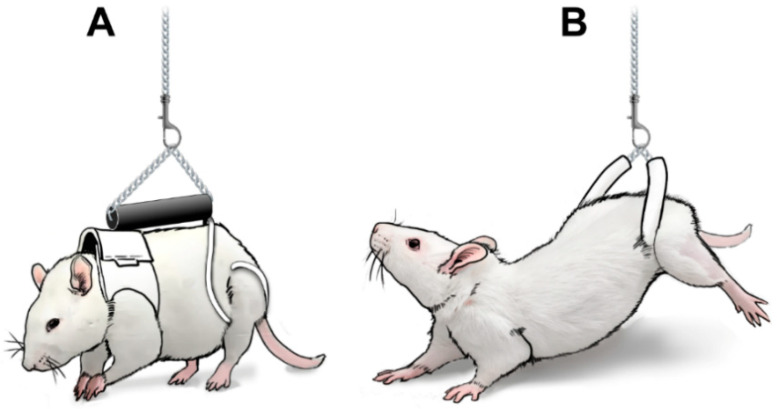
Drawings representing the use of the pelvic harness in PWB (**A**) and HLS (**B**).

In both the mouse and rat PWB models, the forelimbs are supported by a fitted jacket [19,20]. In the mouse PWB system, the hind limbs were supported by a bandage wrapped around the base of the tail, based on the initial tail-suspension design developed for ground-based HU experiments [16] in use since the 1980s. While the HU model and the technical aspects of tail-suspension are beyond the scope of this article, excellent reviews have been published elsewhere [36,37,38]. In the rat PWB system, we chose to adapt a pelvic harness to support the hind limbs, in lieu of the traditional tail suspension (described thereafter as HLS) [20] (Figure 3). This pelvic suspension was first described by others in rats undergoing HU [39] and has been shown to result in similar alterations regarding body weight, muscle atrophy, bone loss and glucose homeostasis compared to tail-suspended animals, while reducing lordosis and spine curvature. The pelvic harness was extremely well tolerated in all of our studies involving either PWB or HLS, and allowed animals to be maintained in the same environment while reducing the time needed to transition from one mechanical loading level to another [20,35,40,41].

### 2.4. Reliability

As in the mouse model, reliability of the partial reduction in mechanical loading was assessed daily by recording both the unloaded and fully-loaded body weight. The ratio of these two measurements provides the achieved partial weight-bearing level. Although the target level is established prior to the experiment (i.e., 70% of full-loading PWB70, 40% of full-loading PWB40, and 20% of full-loading PWB20), daily variations in food intake, water intake, and body weight could affect the effective ratio. Using stainless-steel chains, we demonstrated that during a month-long experiment conducted on 149 rats, coefficients of variations did not exceed 1.8% regardless of the targeted partial unloading level [40]. This represented a marked improvement compared to the initial mouse model. Indeed, whereas the first mouse model design (based on a spring) succeeded to remain within a 5% error margin of the desired unloading level only 77% of the time over a 21-day experiment [19], the rat suspension design (based on a chain) was extremely reliable and allowed for the animals to remain within ± 5% of the desired unloading levels 100% of the time during 28-day periods [20].

## 3. A Global Model to Study Physiological Alterations

### 3.1. Behavior

Animals’ behavior is a great indicator of stress and tolerability [42]. In an effort to reduce stressful situations and better assess animals’ abnormal behaviors, every experimenter involved in animal care engaged in hands-on contact with each rat, in the week preceding baseline. Increased knowledge of animals’ individual behavior and reactions was critical for ensuring proper care and behavioral assessment during the study. Starting on the day of acclimation to the custom housing environment, each animal was monitored by the experimenter once or twice per day, and constant video surveillance was used within the animal facility to observe and understand nocturnal behavioral changes. Daily clinical assessment notes were logged, and health monitoring involved several parameters that are summarized in Table 1.

HU usually results in a transient weight loss in both mice and rats [23,39,43,44]. In the mouse PWB, it has been observed that females lose weight during the first week of partial unloading but are able to return to their baseline body weight after 21 days of exposure [19,21]. In rats, we initially reported a transient weight loss during the first week of partial unloading, regardless of the PWB level [20,35,41]. Adult male rats between the ages of 10 and 30 weeks display a steady growth of the musculoskeletal system which is associated with a constant increase in body weight [32]. This continuous growth was seen in our fully loaded controls (PWB100), and all suspended rats eventually recovered, and exceeded, their pre-suspension body weight regardless of their PWB level [20,40]. However, our latest studies failed to demonstrate a significant difference in body weights across all groups, from 20% to 100% of normal loading during a 28 days experiment [45].

When first exposed to PWB, animals usually display a significantly lower food consumption during the first 48 h, especially when assigned to PWB20 or PWB40 groups [20]; however, cumulative food intake typically remains identical across all groups throughout the experiment [20,41,45]. Both body weight and food intake decrease can be signs of discomfort and stress, which is why daily assessment remains crucial when using the rat PWB model. Animals should be removed from the experiment when experiencing weight loss greater than 15% of their pre-suspension value [39]. Additionally, because the cages do not have as much bedding compared to a traditional rat cage and singly housing the rats prevents body heat transfer between animals, care should be taken to ensure appropriate temperature of the housing facility as alterations to core body temperature can influence overall metabolism [46,47,48], such as the use of cotton for nesting.

Unloading experiments can, and have been completed by assessing several plasma parameters such as blood glucose and corticosterone, both known to increase significantly in response to stressful conditions through the activation of the hypothalamic-pituitary-adrenal (HPA) axis [49,50,51]. As both of these parameters are regulated by the circadian rhythm, particular caution was given to the blood collection time and duration [52,53]. For fully-loaded animals, we showed that the full harness did not elicit a stress response during a 28 day experiment, and that reduced mechanical loading did not impact plasma glucose and corticosterone [45] at 7, 14, and 28 days of exposure compared to pre-suspension values. This finding was further validated by assessing the wet mass of several organs (i.e., adrenal glands and spleen), known to change in stressful conditions. These results represent the first evidence for the lack of chronic stress in the rat PWB model; however, future attention should be paid to the acute response in the first 48 h of exposure to decreased mechanical loading.

### 3.2. Muscular System

The rat PWB model was first established in order to determine the longitudinal time-course of the musculoskeletal impairments [20,40,54]. Indeed, spaceflight and mechanical unloading are known to lead to a rapid decline in muscle mass and function, which in turn increases the risk of fractures, chronic pain, fatigue, and injury upon return to normal loading [55,56,57,58,59,60]. Previous ground-based animal models were extremely useful to obtain a clear picture of the physiological alterations and associated mechanisms, which helped the development of mitigating countermeasures for space crews such as exercise [61,62,63,64], the use of bisphosphonates [65,66], and nutraceuticals [67].

In response to microgravity and mechanical unloading, postural muscles such as the soleus atrophy quickly in humans [55,56,64,68,69] and rats [39,70,71,72,73,74]. Using the rat PWB model, we relied on both indirect and direct measures to determine the alterations in muscle size, force, and function, during exposure to 20% to 100% of full-loading [20,35,40,41]. Calf circumference was used as a weekly indirect indicator of the triceps surae (i.e., soleus and gastrocnemius) size. We demonstrated that exposure to PWB20, PWB40, and PWB70, resulted in a significantly smaller limb girth compared to the PWB100 controls at all times [20,40,41] (i.e., 7, 14, and 28 days of PWB). Additionally, we showed that rear paw grip force either failed to increase or displayed a net decrease after exposure to all levels of PWB, with a linear relationship between gravitational load and muscle impairments [20,35,40]. These force measurements obtained voluntarily were further confirmed by applying a tetanic nerve stimulation and recording the maximum torque of the foot during either plantar or dorsi-flexion [40]. By doing so, we observed a significant decrease in the maximal torque obtained that was linearly correlated to the degree of mechanical unloading.

**Figure 4:**
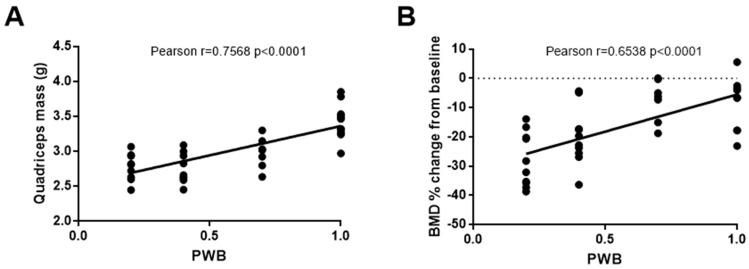
Correlations of muscle mass or bone mineral density with PWB level. Correlation between the quadriceps wet mass and the PWB level after 14 days of exposure (**A**), and between the change in trabecular bone mineral density (BMD) and the PWB level after 14 days of exposure (**B**). Lines represent the best linear fit and Pearson r’s correlation values and significant are displayed on the graph. Adapted from reference [20].

Hindlimb muscle mass revealed the presence of a significant atrophy linearly correlated to the PWB level as soon as 7 days of exposure, our earliest time-point, and until 28 days of exposure [20], [35,40,41] (Figure 4A). This muscle atrophy was further associated with a decrease in myofibers cross-section area (CSA) in both the soleus and gastrocnemius. In humans and rats, the soleus, a postural muscle [75,76] is mostly comprised of fibers expressing the type 1 myosin heavy chain (MyHC1), which generally act as slow-twitch oxidative fibers providing endurance. During disuse and total mechanical unloading, soleus myofibers have been reported to switch from type 1 to type 2 (i.e., generally fast-twitch glycolytic) [77,78,79,80], comparatively more prone to fatigue [81,82]. We also see this modification in soleus composition of rats undergoing reduced mechanical loading for up to 28 days, with 40% of gravitational loading having ~6–7% shift in Type I fiber percentage (Supplementary Tables) [40,41]. However, we must acknowledge that up to this point we have only investigated MHCI fibers and not the separate isoforms of type II MHC fibers, which may have different functional implications [83]. As such, future works will be necessary to investigate the nuances of different MHC isoforms and PWB. Taken together, our data demonstrated that partial mechanical loading results in a dose-dependent muscular deconditioning in the hindlimbs, consistent with the physiological alterations observed during spaceflight [56,74,84,85,86] and hypokinesia [39,73,87,88].

In space, studies usually focus on the calf muscles as astronauts’ leg muscles atrophy quicker than their arm muscles due to their normal bipedal stance. However, animal models are traditionally developed in quadrupedal rodents. Henceforth, one of the advantages of using the PWB model instead of the traditional HLS is the possibility of investigating both the fore- and hind- limbs muscle in response to partial mechanical unloading. Recently, we used the rat PWB model to investigate if exposure to PWB20, PWB40, or PWB70 for 28 days led to a significant muscle deconditioning in the forelimbs compared to fully-loaded animals (PWB100). While we previously have shown that front paw grip force is impacted by partial mechanical unloading [41], we established for the first time that the biceps brachii displayed a 26% reduction in mass during partial unloading, regardless of the level [45]. However, in the triceps brachii, we showed that atrophy was linearly correlated to the level of PWB, with reductions ranging from 9% to 17% compared to the controls. Histomorphometric analysis yielded similar results with a dose-dependent reduction in myofiber CSA. This quadrupedal modeling of partial gravity may allow for a more thorough investigation on the impacts of partial and microgravity on non-weight bearing tissues, as recent works have suggested that non-weight bearing bones can be impacted by spaceflight months after returning to Earth [89].

### 3.3. Skeletal System

Weightlessness, either real or simulated, has been used extensively to study bone loss and establish countermeasures for astronauts [65,90,91]. Using the mouse PWB model, studies have shown that 21 days of exposure leads to a significant decline in body and hindlimb BMD and tibial trabecular bone volume, that was linearly correlated to the degree of unloading [21,23]. Moreover, it has been shown that even PWB70 did not prevent the emergence of skeletal alterations [25]. Similarly, in rats, we demonstrated that 2 weeks of PWB led to a dose-dependent and linear decrease in tibial BMD [20] (Figure 4B). Further studies were conducted to establish the longitudinal time-course of skeletal impairments during partial mechanical loading and established the existence of a potential threshold for the preservation of the skeletal system [54]. While trabecular BMD declines within 7 days of exposure to PWB, it further decreases over a month-long experiment, regardless of the PWB level, suggesting that a plateau was not reached. These results obtained in vivo were further validated by the ex vivo analysis of the distal femoral area via microcomputed tomography. Indeed, our results highlighted a dose-dependent decrease in bone volume, BMD, and trabecular number [54]. Quantitative histomorphometry using calcein staining emphasized that weakening of the trabecular bone architecture during partial mechanical unloading was partly due to decreased osteoblast surface area and activity while osteoclasts remained similar compared to controls. These findings are in agreement with prior research utilizing the HU model and provide further validation for this model [92,93,94,95].

### 3.4. Cardiovascular System

The cardiovascular system is severely impacted during weightlessness [9,96,97,98,99,100], in particular, orthostatic intolerance appears to be common among astronauts upon returning to earth. Many studies have been conducted in the HU model [101,102,103,104,105,106,107], taking advantage of the 30 degree head-down-tilt designed to induce a cephalic fluid shift. Some of these HU models found hypovolemia, altered nitric oxide signaling, and altered sympathetic neurological activity [105,106,108], which may contribute to cardiovascular complications during spaceflight. However there remains controversy on how each of these mechanisms contribute to cardiovascular complications [104]. As the rat PWB is designed to allow quadrupedal unloading, it is not associated with a fluid shift. However, until recently, the impact of partial mechanical unloading on the cardiovascular system including the effects of the pelvic harness in lieu of the tail suspension, had not been addressed. Therefore, we longitudinally monitored tail blood pressure, foot oximetry, and heart rate for 28 days in all conditions [45]. Our results highlighted that the use of a pelvic harness during normal loading did not impair indirect measures of blood flow nor did it modify blood pressure and heart rate over the course of the experiment. This study further demonstrated that partial reductions in mechanical loading did not reveal significant changes in these parameters. While these results are preliminary, they may suggest cardiovascular protections may be conferred with minimal gravitational force, such as those found on extra-terrestrial targets such as the Moon or Mars with 1/6th and 1/3rd of the Earth’s gravitational pull. However, more works using PWB are necessary to understand the nuanced cardiovascular mechanisms that may contribute to potential protection from spaceflight-induced orthostatic intolerance.

## 4. Future Perspectives

### 4.1. Investigating Shifts Lower than 1g

The PWB rat model was designed to be adaptable to any mechanical loading ranging from HLS to PWB100 (i.e., total mechanical unloading of the hind limbs to full mechanical loading on all limbs). Such design has allowed us to investigate gravity as a continuum and mimic gravity shifts lower than 1*g*. For example, astronauts en route to extra-terrestrial targets such as the Moon or Mars, will inevitably be exposed to a first phase of microgravity. In 2019, we developed a new paradigm by exposing animals to either two weeks of PWB40, or one week of HLS followed by one week of PWB40 [35]. This was made possible by the seamless transition between the two systems and allowed us to assess the consequences of partial mechanical loading on an already compromised musculoskeletal system.

**Figure 5:**
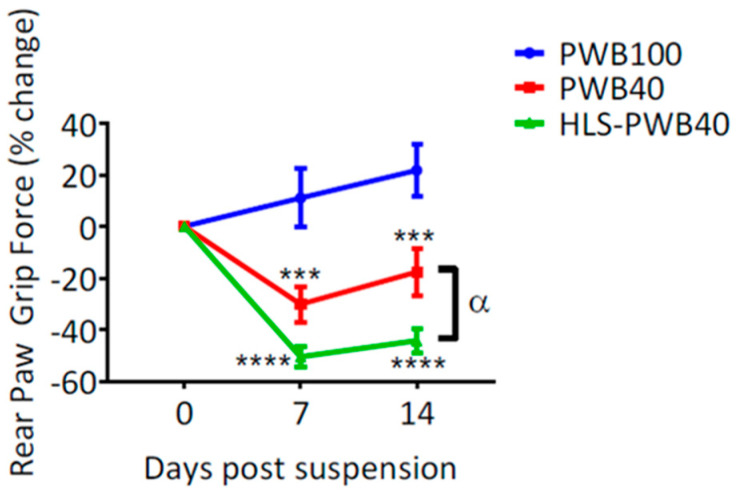
Changes in rear paw grip force. Changes in rear paw grip force compared to pre-suspension values (day 0) in rats undergoing 14 days of normal loading (PWB100), 14 days of loading at 40% (PWB40), and 7 days of hindlimb suspension immediately followed by 7 days of loading at 40% (HLS-PWB40). Results are displayed as mean ± SEM and were analyzed with 1-way ANOVA and Tukey’s post hoc test and are represented as ***, ****: *p* < 0.001 and *p* < 0.0001 vs. PWB100, α: *p* < 0.05 PWB40. Adapted from reference [39].

We demonstrated that 7 days of HLS led to a significant loss of muscle force in the hind limbs, greater than what was observed after 7 days of PWB40. Once exposed to an increased mechanical load, animals’ grip force remained significantly lower than the PWB40 group (Figure 5). Furthermore, muscle wet mass was lower in the triceps surae. These preliminary data reveal that investigating partial gravity environments on healthy animals might not depict an accurate picture of what could happen in space. While we did not investigate other physiological systems in such a regimen, it is necessary to better understand how animals, and humans, whose bodies are already adapting to weightlessness, will react to shifts in gravitational environments.

Using the rat PWB model also provides the option to investigate the various stages associated with lunar spaceflight (e.g., microgravity en route, partial gravity on the lunar surface, microgravity during return travel, reloading upon return to Earth). So far, it has been essentially impossible to investigate the effects of weightlessness without exposing the animals and astronauts to a significant period of hypergravity during re-entry. This powerful and sudden increase in mechanical loading has been known to rapidly impact the musculoskeletal system and is described as a reloading injury [61,71,109,110,111,112]. Although more experiments are designed with an end point in flight to avoid this injury, data remains scarce and little is known about the effects of reloading injury compared to microgravity. In the near future, we will make it a priority to use the PWB model in order to accurately mimic space missions and investigate each stage, i.e., total unloading, partial loading, partial reloading, and total reloading. These data will also be critical in order to better understand how effective artificial gravity treatments could be in mitigating physiological alterations during spaceflight.

### 4.2. Mechanisms Contributing to PWB Phenotypes

Years of research using the full HU model has found robust inductions of catabolic signaling cascades such as the protein ubiquitin and autophagy pathways [113,114,115,116]. Our current PWB model suggests gravitational dose-dependence with regard to muscle size; however, we have not yet investigated if the same degradative pathways are activated during PWB or if these same gravitational dose-dependence exist between catabolic pathways. Understanding cellular signaling contributing to the PWB phenotype will provide a more nuanced understanding of this model and how to best maximize PWB to develop effective strategies for astronaut health, both in space and upon returning to Earth.

### 4.3. Sex-Based Differences

The rat PWB model was developed using adult male rats, chosen to represent the predominantly male astronauts’ population. However, the astronauts’ population remains very heterogeneous in sex, age, mission duration, and mission protocols. More so, it is clear males and females have different musculoskeletal [117,118,119], cardiovascular [120,121] and overall physiology that may contribute to differential responses to micro- and partial gravity. As such, it is of the utmost importance to study adult female animals, in order to understand the sex-based differences in the adaptations to partial gravity, and thus develop the best countermeasures for both males and females.

We recently exposed adult female rats of the same age and strain, to 14 days of normal-loading using a full harness (PWB100) or 14 days of PWB40 [122]. In order to accurately validate our model in female rats, we extensively assessed hindlimb blood oxygenation, stress, and food intake. None of these parameters revealed differences between the two PWB groups and confirm what we previously reported in male rats [45].

Contrasting male rats, female rats appeared to be quite resistant to PWB by maintaining their body weight constant and not displaying a loss of muscle force during the first week. However, on day 14, females exposed at PWB40 displayed a significant decrease of rear paw grip force compared to their baseline, which was associated with a significant atrophy of the gastrocnemius muscle. Surprisingly, the soleus did not exhibit significantly smaller wet mass compared to the controls at day 14; though there was significant atrophy of the type I myofibers. Regardless, these preliminary works may suggest differential responses to PWB in males and females and warrants further direct investigation.

While we did not establish the time-course of muscle alterations and only investigated one partial unloading level at 40% of full-loading, these results represent the first step required to begin to understand the sex-based differences that exist in response to a decreased mechanical load, and successfully develop mitigating strategies for the female astronauts embarking on lunar missions.

### 4.4. New Therapeutic Countermeasures

To date, only one countermeasure has been tested using the rat PWB model. Indeed, we recently assessed the benefits of a moderate daily dose of resveratrol in male rats exposed to partial mechanical unloading at 40% of their normal weight-bearing (PWB40, Martian gravity analog) [41]. Resveratrol (or trans-resveratrol, RSV) is a polyphenol commonly found in the diet [123,124], more specifically in grape skin, blueberries, and peanuts. RSV has been studied in both animals and humans, and is associated with a wide range of biological effects and cellular pathways. Amongst its properties, it has been shown that RSV acts as an anti-inflammatory [125], anti-oxidant [126], anti-diabetic [127], as well as an osteoprotective agent [128,129] and exercise mimetic [129,130], making it an exceptional candidate to help mitigate muscle deconditioning in our partial gravity analog model. We find that a moderate does of RSV appears to blunt reductions in front and back paw grip force as well as partially protect myofiber cross-sectional area in both the soleus and the gastrocnemius [41]. Unlike the studies conducted in animals fully unloaded, we did not treat animals with RSV before exposure, thus assessing if RSV could prevent muscle disuse when given concomitantly. Many other potential therapeutic interventions have been tested in the traditional HU model including: protein supplementation [131,132], various antioxidants [133], allopurinol [134] (pharmaceutical used for treating gout), heat stress [135,136], 8-Prenylnaringenin [137,138] (a prenylflavanoid derived from hop extracts), testosterone [139,140], and others. Many of these interventions showing limited success for attenuating HU-induced muscle loss. While some of these interventions in themselves may not fully protect muscle atrophy, there is the possibility for an interaction between partial gravity and an additional intervention, which should not be understated.

## 5. Outstanding Questions

### 5.1. Circadian Rhythm

While the PWB model helped address many physiological aspects due to reduced mechanical loading, more experiments are required in order to obtain a more complete picture of the hazards encountered by the astronauts who will undertake the Artemis missions. Circadian rhythmicity is the eukaryotic process that regulates physiological functions with a ~24 h cycle. While the suprachiasmatic nucleus is the master clock, many organs display a secondary clock that can, in some cases, be asynchronous due to the shift of important cues (e.g., change in feeding time can alter the stomach internal clock [141]). It has been established that circadian rhythms play an important role in many physiological processes on the ground including sleep cycle [142], gut microbiota [143], energy metabolism [144], heart rate and blood pressure [145,146], and that disturbances in our innate rhythm can lead to obesity [147,148,149], diabetes [148], and fertility complications [150]. Onboard the international space station (ISS) astronauts can witness 16 sunrises and sunsets every day, however, indoor lighting is used as a mitigating strategy [151] and simulates a 24-h cycle within the habitat. Astronauts report sleep disturbance more frequently in-flight than on the ground [152,153,154], and sleep deprivation has been associated with a decline in crew performance [153] and a change in alertness levels [152,155]. Moreover, the cardiovascular system is impacted by circadian rhythm and spaceflight leads to significant alterations in heart rate [156,157], blood pressure [158] and cardiovascular rhythms [159]. Similar observations have been made in animal models [160,161,162] and in human ground-based analogs such as the 520-d Mars mission [163]; and sex-based differences have been observed between astronauts [164]. Upon arrival on the Moon and Mars, astronauts will experience yet new light-dark cycles, and new ground-based models should now investigate the coupling effect of reduced loading and altered circadian rhythms to develop new and effective countermeasures.

### 5.2. Radiation

Humans on earth are protected from the most harmful space radiation due to the presence of our magnetosphere and the Van Allen belts [165]; however, astronauts receive a higher dose of solar particles. This radiation exposure to galactic cosmic rays (GCR) is problematic for astronauts on low-Earth orbit space (LEO) missions (e.g., missions carried onboard the ISS), but this exposure may be particularly impactful during long duration missions, such as to the Moon or Mars It has long been known that radiation has deleterious effects on physiological systems [69] including on the skeletal [26,67,166,167], muscular [168], neurological [168,169], immune [170], and cardiovascular systems [96,106,171]. More recent works have begun to establish that radiation and microgravity are associated with a shift in circulating miRNA expression [172]. Taken together, the aggregate of evidence strongly implies radiation in combination with microgravity has the potential for additive effects on human physiology. However, animals in ground-based analog studies are often exposed to an acute dose of radiation meant to represent the total amount received during a potential journey, and often exposed to single or dual beams, which limits our understanding of the short-term consequences of radiation for astronauts who will undertake journeys to the Moon and Mars. As such, future studies will need to investigate the influence of radiation and partial gravity on physiological outcomes. The rat PWB model will allow researchers to assess the coupled effect of mechanical loading and radiation (i.e., the mechanical loading of the Moon coupled with radiation environment on the lunar surface). Moreover, our cages can easily be made radiation-safe (i.e., be used to house animals during exposure without creating artefacts) and could be useful to expose animals to daily doses of radiation and PWB simultaneously.

### 5.3. Biomarkers

Starting with the Artemis missions and to Mars thereafter, astronauts will not have access to the extensive exercise [61] nor the medical equipment currently present on the ISS; thus we need to find reliable biomarkers to assess astronauts’ conditions in flight. The recent development of –omics and the public availability of numerous data sets from NASA GeneLab [173], allowed for some new discoveries including the importance of miRNAs. MiRNAs are small non-coding RNA molecules that are largely conserved throughout species and act as RNA-silencer, thus able to regulate post-transcriptional gene expression [174]; and can be associated with pathological conditions including cancer [175,176,177], diabetes [178,179], cardiovascular diseases [180], and viral infection [181]. Recent work has shown that both real and simulated spaceflight lead to an increased expression of several circulating miRNAs, now considered as a space-signature [172,182], and that antagomir supplementation could constitute preventive countermeasures for astronauts.

Regarding muscle health, electrical impedance myography (EIM) has been widely used in both animal and human models to assess muscle quality and disuse [183,184,185] and detect muscular diseases [186,187,188]. EIM is a small, non-invasive tool that uses electrical current to obtain muscle properties at various frequencies in a few seconds, and can be coupled with ultrasound, a tool that will most likely be made available for astronauts, for increased potency [189,190,191]. Recently, it has been shown that EIM can discriminate between muscle atrophy and loss of function in response to total unloading and in response to partial weight-bearing [192], making it a valuable tool for astronauts that will be exposed to both microgravity and partial-gravity during their mission.

## 6. Conclusions

In conclusion, the PWB model is a novel experimental method that has extensive potential for the investigation of physiological alterations in response to both micro- and partial gravity. We have thus far been the first group to create and utilize the PWB in rats, both males and females, but anticipate we will not be the last. With this review we hope to encourage other biomedical investigators to adopt this model within their own laboratories. A combined research effort from many research laboratories will be imperative to thoroughly understand the complex physiology of spaceflight. More so, a broader understanding of spaceflight physiology may also facilitate important discoveries of human physiology that can benefit ground-based medicine. Regardless, to ensure the safety of astronauts in future missions to the Moon, Mars, and beyond, we need to first understand the complex interactions between human physiology and spaceflight, and the PWB model will play a critical role in unraveling these nuanced relationships.

## Figures and Tables

**Table 1:** List of daily assessments recorded for each animal throughout the entire study.

Visual Assessments (Hands-Off)	Clinical Assessments (Hand-On)
Integrity of the harness, jacket, suspension apparatus	Body weight, unloaded weight, achieved PWB
Posture	Porphyric staining
Alertness and ability to walk	Fur coat appearance around jacket and harness
Food intake	Presence of skin irritation, redness or abrasion
Water intake	Efficient grooming of the genital area (evidence of sperm plugs, vaginal secretions)
Presence and consistence of feces	Appearance of the teeth
Evidence of grooming	Edema
Use of all limbs to balance	Evidence of broken nails/bleeding
